# Lessons learned to improve COVID-19 response in communities with greatest socio-economic vulnerabilities

**DOI:** 10.1186/s12889-023-15479-0

**Published:** 2023-04-06

**Authors:** Payam Sheikhattari, Rifath Ara Alam Barsha, Emma Shaffer, Istiak Bhuyan, Bethtrice Elliott

**Affiliations:** 1 School of Community Health and Policy, Prevention Sciences Research Center, Morgan State University, 1700 East Cold Spring Lane, Baltimore, MD 21251 USA; 2 Center for Urban Health Disparities Research and Innovation, Morgan CARES Community Engagement Core, Morgan State University, 1700 East Cold Spring Lane, Baltimore, MD 21251 USA; 3 Department of Transportation and Urban Infrastructure Studies, Morgan State University, 1700 East Cold Spring Lane, Baltimore, MD 21214 USA; 4 Center for Urban Health Disparities Research and Innovation, Morgan State University, 1700 East Cold Spring Lane, Baltimore, MD 21214 USA

**Keywords:** Community Engagement, COVID-19, Geographic Information System (GIS) mapping, Vulnerable Communities, Baltimore City, Social Determinants of Health

## Abstract

**Background:**

Vulnerable communities are susceptible to and disproportionately affected by the impacts of the COVID-19 pandemic. Understanding the challenges faced, perceptions, lessons learned, and recommendations of the organizations that provide services in response to COVID-19 to vulnerable communities is critical to improving emergency response and preparedness in these communities.

**Methods:**

This study employed GIS mapping to identify the needs and assets that exist in communities in Baltimore City, where vulnerabilities related to social determinants of health and the burden of the COVID-19 pandemic were greatest. We also conducted an online survey between September 1, 2021, and May 30, 2022, to assess the COVID-19-related services provided by local organizations, challenges faced, perceptions, lessons learned, and recommendations to inform policies, programs, and funding related to improving the COVID-19 response in underserved communities. The survey was disseminated through the online Kobo Toolbox platform to leaders and representatives of organizations in Baltimore City.

**Results:**

Based on GIS mapping analysis, we identified three communities as the most vulnerable and 522 organizations involved in the COVID-19 response across Baltimore City. 247 surveys were disseminated, and 50 survey responses were received (20.24% response rate). Out of these organizations, nearly 80% provided services in response to COVID-19 to the identified vulnerable communities. Challenges experienced ranged from funding (29%), and outreach/recruitment (26%), to not having access to updated and accurate information from local officials (32%).

**Conclusions:**

This research highlights critical insights gained related to the experiences of vulnerable populations and suggests ways forward to address challenges faced during the emergency response by providing recommendations for policy and program changes. Furthermore, the findings will help better prepare vulnerable communities for public health emergencies and build more community resilience.

**Supplementary Information:**

The online version contains supplementary material available at 10.1186/s12889-023-15479-0.

## Background

The outbreak and rapid spread of the novel coronavirus (COVID-19) pandemic became a global health problem due to its high transmission rate and swift development worldwide [1]. Within the first few months of its spread, beginning in December 2019 in Wuhan, China, the number of cases drastically increased and reached nearly 80 million cases within one year in December 2020, with over 1.7 million deaths globally [2][3]. With more than 93 million confirmed COVID-19 cases and over one million associated deaths as of August 2022, the United States is one of the most impacted countries (Centers for Disease Control and Prevention, 2022). Although the pandemic may have affected most people across the United States in some capacity, disease surveillance reports documented COVID-19 disproportionately impacting individuals and families in vulnerable communities, specifically racial and ethnic groups such as African American, Spanish speaking and immigrant populations residing in low-income communities [4, 5]. Vulnerability is a term that describes communities facing pre-existing disparities in social determinants of health including health and quality health care, neighborhood environment, food access, education, transportation, and economic stability due to inequalities that challenge a community’s ability to cope with disastrous outbreaks such as the COVID-19 pandemic [6–8]. Numerous reports suggest that people residing in vulnerable communities disproportionately had greater risks of infection, worse health outcomes, hospitalizations, and death from COVID-19 [9–11].

Previous studies highlight pandemic adverse outcomes and challenges. Uncoordinated approaches in vulnerable communities shed a harsh light on the need for better policy solutions to improve integrated COVID-19 prevention services, particularly in vulnerable communities experiencing myriad health inequities, now exacerbated by COVID-19. Despite advances and improvements in health care and disease prevention, with gaps in health coverage, Black communities are left susceptible to environmental factors, leading to poorer outcomes. At the beginning of the pandemic, the lack of transportation to testing sites was another issue [6]. In large cities, testing sites were more prevalent in economically stable communities compared to the poor neighborhoods. Moreover, residents from low-income neighborhoods with COVID-19 symptoms were less likely than well-off individuals with the same symptoms to receive a COVID-19 test [9–11]. After this disparity became public knowledge, agencies began to work to protect the most vulnerable populations by concentrating COVID-19 testing sites in areas where these individuals lived and had access, such as in churches and nearby community centers. To ensure a timely response to COVID-19 and other future outbreaks, the availability of testing sites, access to health care systems for treatment, and the interpretation and reporting of findings need to be strengthened in vulnerable communities [12]. While healthcare centers provide vital services, non-health organizations like faith-based groups, schools, and charities also play a critical role in meeting the needs of vulnerable groups through education, financial assistance, and other support services.

As the largest city in Maryland and the 22nd most populous city in the U.S., Baltimore has a population of around 585,708 people - over 60% identifying as Black or African American, and approximately 29% as White. Baltimore’s demographic profile is similar to other urban cities with significant African American populations like Detroit, Philadelphia, and Atlanta. However, the city faces challenges related to poverty, crime, and population decline, which has prompted ongoing revitalization efforts [13]. Because of the strikingly high prevalence of long-standing social, environmental, and financial challenges, communities in Baltimore, specifically ethnic and racial groups, were disproportionately impacted by COVID-19 in terms of higher mortalities and hospitalizations as well as other indirect burdens such as lost income, etc. [14, 15]. Existing challenges in Baltimore present large populations of families residing in low-income economically segregated neighborhoods with limited opportunities for high-quality education and decent employment, and access to fresh food and quality health care [16, 17]. The majority of residents have a great dependence on public transportation to access parts of the region where jobs, healthier food, health care, and better education are located, and the ease in transit accessibility and length of distance to these destinations is limited [14, 17, 18]. In addition, the pandemic and the related shutdown of support services, such as public transportation further exacerbated the struggles for many residing in underserved communities [19]. A recent collaborative study between Johns Hopkins University, the Baltimore Transit Equity Coalition (BTEC), and Baltimore community members examined public transportation and health impacts in communities in Baltimore [20]. Utilizing publicly available data and geographic information systems (GIS) tools, data pinpointed vulnerable communities in greater need of better transportation and highlighted recommendations for highest need areas [21].

This study uses GIS technology to demonstrate community socio-economic vulnerabilities, pandemic response, and input from community stakeholders in Baltimore. Given the impact of COVID-19, the future waves, the rise of deadly variants, and the appalling death tolls, it is important to identify and ensure a timely and equitable administration of medical care, health services, and essential resources for all individuals in affected communities. To overcome these limitations, successful emergency response requires identifying and managing locations or facilities accessible to the entire population to deliver basic needs and health services at these points. Thus, the purpose of this study is: 1) to identify vulnerable communities in Baltimore City where the burden of the COVD-19 Pandemic was the greatest, 2) to identify and engage with organizations involved in COVID-19 response in the identified communities, and 3) to gain valuable insights into the challenges, perceptions, lessons learned, and recommendations to inform policies, programs, and funding related to improving the COVID-19 response in Baltimore City. Our hypothesis is that low-income and underserved minorities are uniquely vulnerable due to the negative impact of social determinants of health, and policy and medical procedures that address their needs can help mitigate these vulnerabilities. We hope this study will enlighten effective ways of reaching, engaging, and supporting vulnerable communities worldwide.

## Methods

### Study setting and design

**Figure 1:**
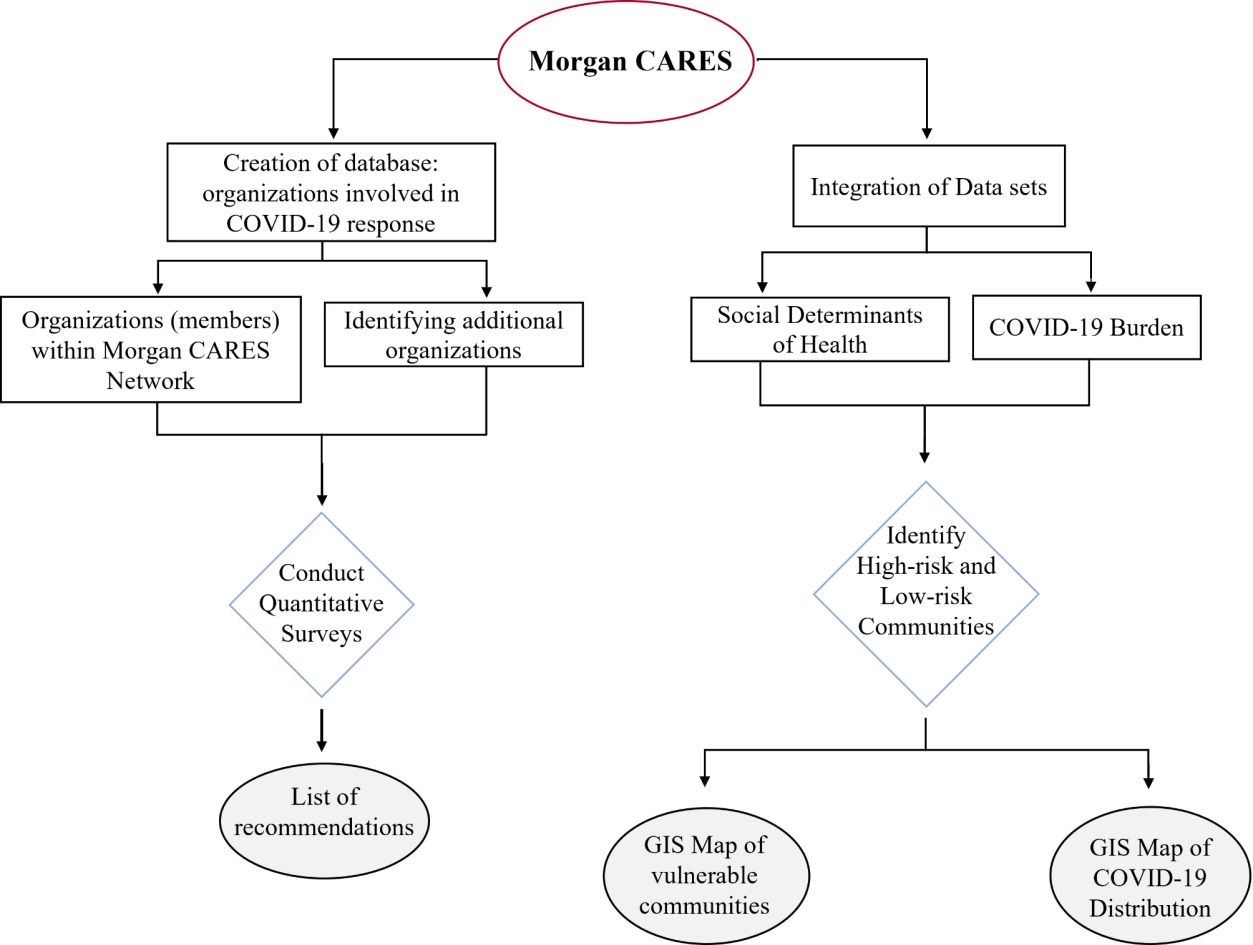
The methodology scheme of the study.

As a part of our ongoing work of community-based participatory research (CBPR), this study’s design, recruitment, data collection activities, and analyses took place at Morgan CARES (Community-Aligned REsearch Solutions). Morgan CARES is the Community Engagement Core of the Center for Urban Health Disparities Research and Innovation (RCMI@ Morgan) at Morgan State University and has a presence working in Baltimore City communities. As the mission is to support a network of members from diverse backgrounds, create new partnerships, and enhance their capacity for addressing pressing health problems in Baltimore by providing resources and training services, Morgan CARES has recruited over 380 community and academic members in Baltimore City. For this COVID-19 outreach study, we engaged members and representatives of organizations within these communities. The details of the data analysis are presented in the flow chart in Fig. 1.

## Study area

We used Community Statistical Areas (CSAs) as the primary geographic unit. CSAs have been developed by the Baltimore City Health Department, which are statistical units formed by aggregating census tracts [22]. The resulting 55 CSAs represent a neighborhood of similar socio-demographic and economic characteristics. Each CSA had a total population ranging from 5,000 to 20,000 persons and allows for collecting and aggregating a wide range of data for relatively stable geography over time (Baltimore City Health Department, 2018).

## Data sources

We used data collected through several publicly available databases. The Baltimore City data were obtained from the 2017 Neighborhood Health Profiles. Baltimore City Data compiles a variety of individual and community-level sociodemographic data as well as several health outcomes data from several sources (Baltimore City Health Department, 2017) 23]. COVID-19 data (cases and vaccination rates) were obtained from the Maryland Department of Health beginning in 2021. These datasets are publicly available (Open Maryland Portal) at the zip code level. The sociodemographic data were retrieved from the American Community Survey (ACS) from Census Bureau, which releases new data every year and is publicly available to access with different data tools. Spatial data were retrieved from Open Streets Maps which provides map data such as City boundary, CSA boundary, and the list of organizations used for this study. Geography CrossWalk was also utilized to build relationships and harmonize tract data between different geographic locations for COVID cases retrieved both at the zip code and CSA levels.

To capture the variability of health, social, and economic factors across Baltimore, we developed the COVID-19 Community Vulnerability Index (CCVI). The CCVI is similar to the Social Vulnerability Index, previously developed by the CDC to support disaster management [20], except with additional elements specific to the vulnerable communities impacted by the COVID-19 pandemic. The index combines indicators of vulnerability from four themes (socioeconomic status, accessibility, health, and COVID-19). We defined the following quantities, where $n$ represents the total number of CSAs, *and i* represents the value for the selected indicator at the time (*t*). S represents socioeconomic status, H represents health, A represents accessibility, and C represents COVID-19.

$$CCVI=\underset{i,t}{\overset{n}{\int }}(S+A+H+C )$$

The variables were aggregated at the CSA level. For each CSA, its percentile rank was calculated comparing all CSAs in the city for eleven individual variables, four themes, and its overall position: 1) Socioeconomic status, including “below poverty”, “unemployed”, “high school diploma”, and “non-white population”; 2) Accessibility including “no car household”, “food desert”, and “access to health care”; 3) Health including “heart disease mortality rate” and “lung cancer mortality rate”; and 4) COVID-19 including “positive cases” and “hospitalized.” For each of the four themes, the percentiles were aggregated for the variable comprising each theme. The summed percentiles for each theme illustrate theme-specific percentile rankings. For the overall CSA rankings, the aggregate for each theme was ordered by the CSAs, and then calculated by overall percentile rankings. We further classified the rankings. CSAs in the top 10%, i.e., at the 90th percentile of values, are given a value of 1 to indicate “high vulnerability”. Tracts below the 90th percentile are given a value of 0. For a theme, the category value is the number of flags for variables comprising the theme. We calculated the overall flag value for each CSA as the number of all variable flags. We mapped the following using ArcGIS software: COVID-19 cases and vaccination rates, COVID-19 vulnerable zip codes, socioeconomic variables, and overlapped vulnerable communities identified by comparing the two maps.

## Data Collection and Analysis

To engage with leaders and representatives from organizations in Baltimore City to assess COVID-19 response in their communities, an online questionnaire survey was prepared using KoboToolbox and disseminated to organizations within our created database, between September 1, 2021**,**May 30, 2022. Out of 522 organizations involved in COVID-19 responses across Baltimore City, 247 were selected based on their role in serving vulnerable communities and being active. These organizations included healthcare centers, faith-based organizations, educational institutions, small businesses, and non-profit organizations engaged in providing testing, vaccination, treatment, information sharing, food distribution, shelter, and other support services. Surveys were distributed to one of the key staff and the leaders of these organizations who could describe their organizations’ roles, share their personal opinions on the challenges faced by affected communities, and provide potential policy recommendations to address these challenges. The questionnaire was intended to gain insight on: 1) the organization’s role in COVID-19 response, 3) to capture any difficulties that the organization experienced in providing services to targeted populations in their community, [3] and to understand their perceptions of the COVID-19 response at the City level. The participants’ perception of the COVID-19 vaccine rollout process in Baltimore City was measured using a five-point scale: 1-Not effective, 2-Slightly effective, 3-Somewhat effective, 4-Very effective, & 5-Extremely effective. The survey was completely voluntary and took no more than ten minutes. All responses were anonymous and kept confidential. Each person that completed the survey received a $15 incentive for their participation. Due to COVID-19 restrictions, we could not reach many of the organizations we originally planned to reach. Data analysis was performed using Stata version 15.1 statistical software to calculate the total number and percentage.

## Results

**Figure 2:**
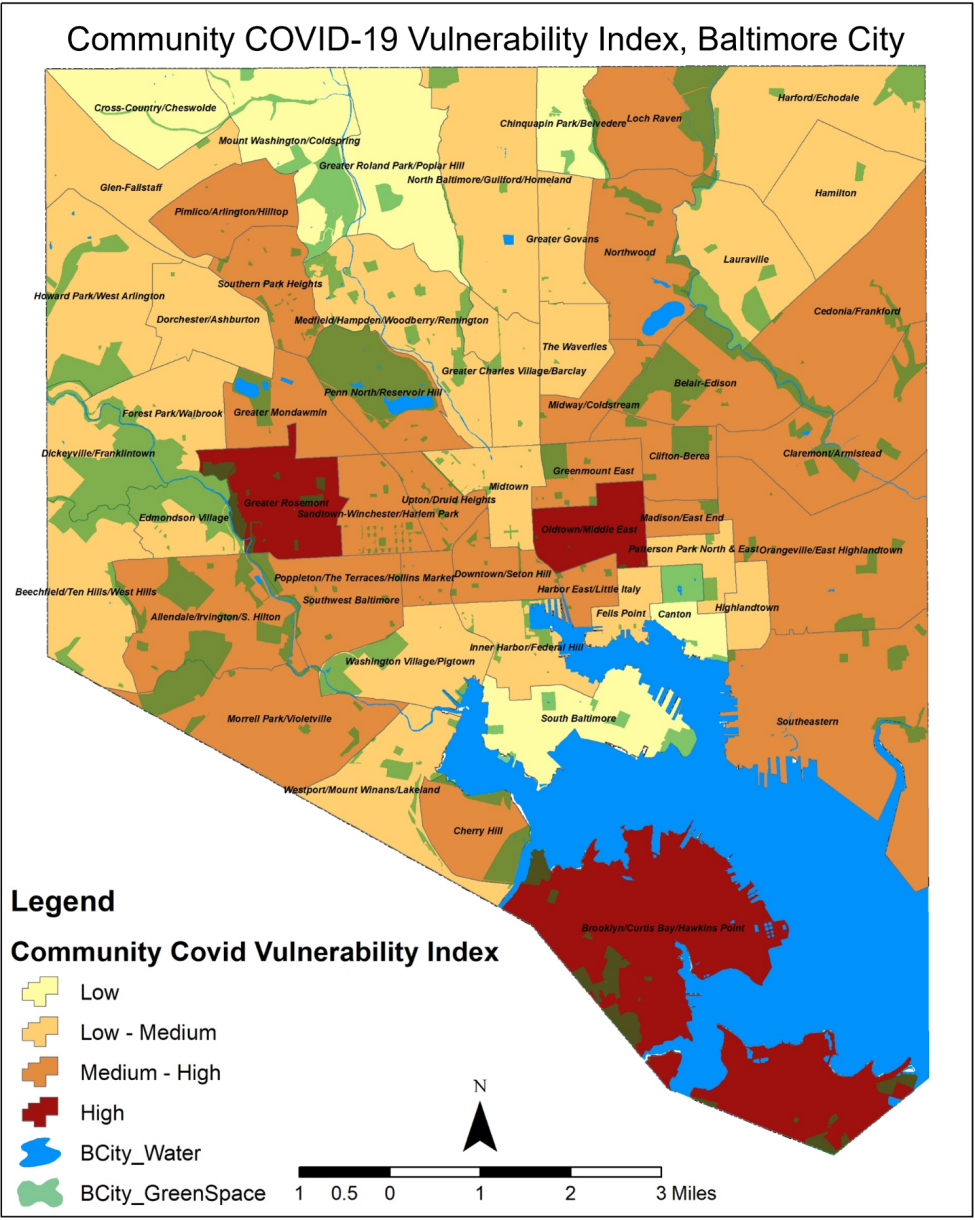
GIS map of Baltimore communities identified as having high or low vulnerabilities based on the community covid vulnerability index.

We examined the neighborhood health profile and the determinants of susceptibility based on social demographics to determine a community’s vulnerability (Supplementary Fig. 1). In further analysis, we captured the variability of health, social, and economic factors across Baltimore utilizing the COVID-19 Community Vulnerability Index. Based on these analyses, 3 CSAs were found to have high vulnerability to COVID-19 risk, 13 had a medium-high vulnerability, 22 were observed to have low-medium, and the remainder of the communities were considered to have less vulnerability during the COVID-19 pandemic (Fig. 2).

**Figure 3:**
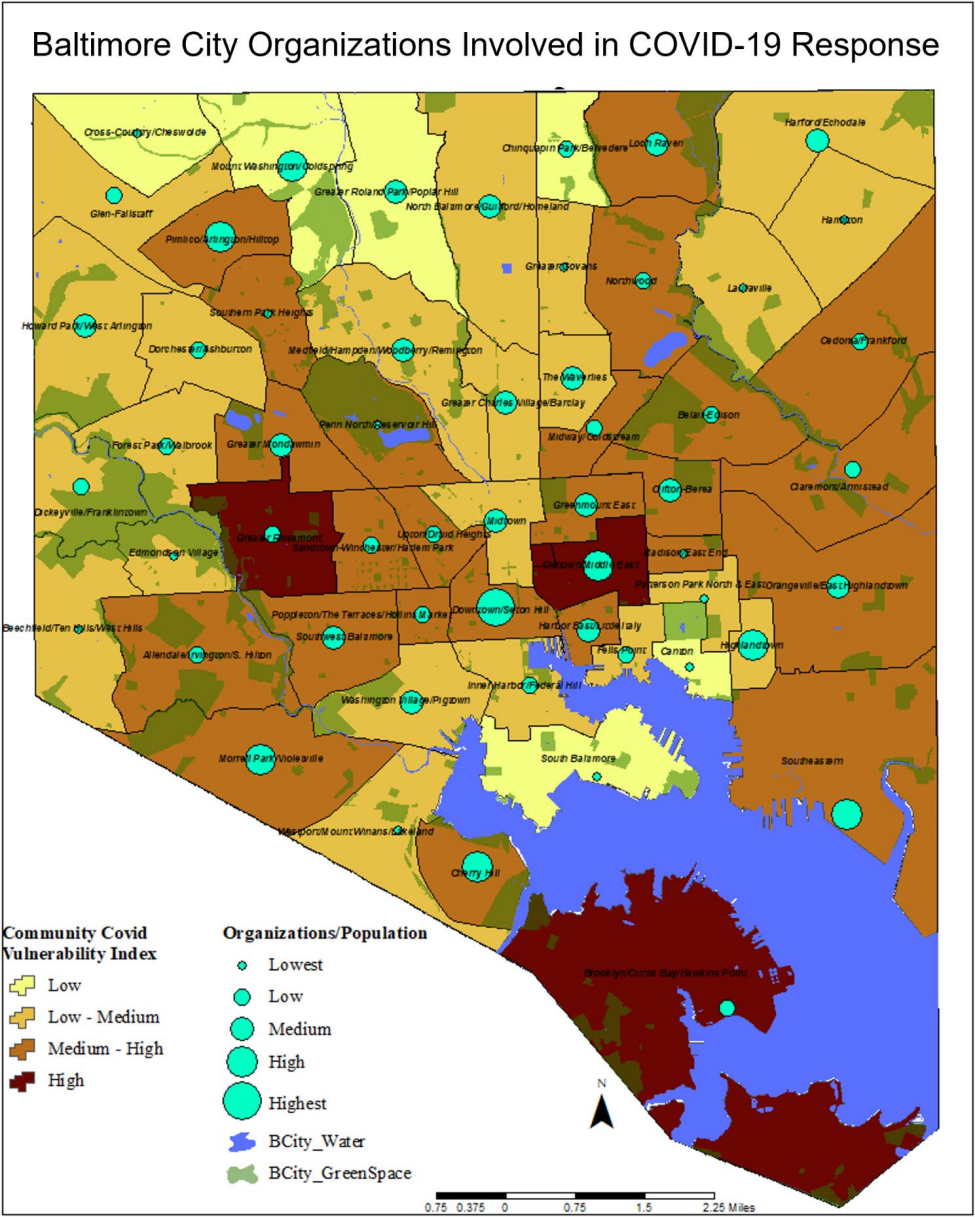
GIS Mapping of organizations involved in COVID-19 Response in Baltimore City.

We identified 522 organizations involved in the COVID-19 response across Baltimore City. To evaluate the organizational response in vulnerable communities, we compared the level of vulnerability with the number of organizations involved. The distribution of organizations was normalized while mapping using ArcGIS software. To perform this, the number of total organizations was standardized against the population data in each of the respective CSAs. Five categories of organization distribution were created including: Lowest (0.0189-0.0465%), Low (0.0466-0.0814%), Medium (0.0815-0.1312%), High (0.1313-0.2154%), Highest (0.2155-0.5581%). Based on these categories, over 200 organizations were involved in the COVID-19 response in low-medium and medium-high vulnerable communities, whereas 32 and 41 were involved in low or high vulnerable communities, respectively (Fig. 3; Table 1).

**Table 1:** Distribution of organizations by community COVID vulnerability

COVID- 19 Community Vulnerability	# of Organizations (*n* = 522)
**Low**	32
**Low-Medium**	201
**Medium-High**	248
**High**	41

We engaged with several leaders and representatives from organizations that have been involved in the COVID-19 response in the identified communities in Baltimore City. Surveys (*n* = 247) were disseminated, and 50 survey responses were received (Table 2). In response to the COVID-19 pandemic, 88% of respondents reported working with community members, serving various populations including children, older adults, persons with disabilities, and the LGBT community. 76% provided COVID-19 related services such as healthcare provider connections, food distribution, and temporary shelter. 30% facilitated access to COVID-related information, 16% provided COVID-19 testing, and 20% served as vaccination centers. 60% of organizations provided both in-person and virtual services, 30% provided in-person only, and 8% provided virtual services only. Half of the organizations formed new partnerships with others during the pandemic. Access to funding (58%) and PPE (24%) were among the challenges organizations faced. Furthermore, only 50% of respondents found the COVID-19 vaccine rollout process to be somewhat effective, with few finding it extremely effective or slightly effective.

**Table 2:** Descriptive statistics of the study sample

Variables	Number of Organizations (*n* = 50)
Working with community members	
Yes	44 (88%)
No	6 (12%)
**Population served**	
Children	29 (58%)
Adolescents and young adults	33 (66%)
Reproductive aged women	27(54%)
Men	29 (58%)
Older adults	30 (60%)
Persons with disability	17 (34%)
Formerly incarcerated individuals	18 (36%)
LGBT community	13 (26%)
Other	10 (20%)
**Mode of communication**	
Phone	33 (66%)
Email	39 (78%)
Posta mail	8 (16%)
Text message	23 (46%)
In-home visits	12 (24%)
On-site conversation	29 (58%)
Virtual meeting	23 (46%)
**Providing services in response to COVID-19**	
Yes	38 (76%)
No	12 (24%)
**Services provided in response to COVID-19**	
Helping those who need assistance connecting with healthcare providers, such as scheduling a vaccination appointment	14 (28%)
Food distribution	21 (42%)
Temporary shelter	1 (2%)
Facilitate access to COVID-19 related information	15 (30%)
COVID-19 testing	8 (16%)
Serving as a COVID-19 vaccination center	10 (20%)
Other	13 (26%)
**Mode of providing services during COVID-19**	
Virtual	4 (8%)
In-person	15 (30%)
Both	30 (60%)
Neither	1 (2%)
**New partnership formation during COVID-19**	
Yes	25 (50%)
No	24 (48%)
Missing	1 (2%)
**Challenges experienced during COVID-19 (Organizational)**	
Funding	29 (58%)
PPE or other cleansing equipment	12 (24%)
Logistical (transportation, facilities, working from home, etc.)	25 (50%)
Employee Illness/Inability to work	12 (24%)
Outreach/Recruitment	26 (52%)
Having access to updated/accurate information from local officials	16 (32%)
Other	1 (2%)
**Challenges experienced by the population served during COVID-19**	
Access to food	27 (54%)
Transportation	37 (74%)
Housing-related issues (eviction, unable to pay rent/mortgage)	26 (52%)
Childcare	19 (38%)
Unemployment/Furlough	20 (40%)
Access to COVID-19 testing site	22 (44%)
Access to COVID-19 treatment site	13 (26%)
Health insurance	16 (32%)
Lack of access to updated/accurate information	19 (38%)
Government response	11 (22%)
Other	0 (0%)
**Effectiveness of vaccine rollout process in Baltimore City**	
Not effective	0 (0%)
Slightly effective	7 (14%)
Somewhat effective	25 (50%)
Very effective	16 (32%)
Extremely effective	2 (4%)

To improve preparedness, the participants recommended better coordination between government organizations, healthcare providers, and non-profit organizations, access to funding, and more consistent and better-executed policies and program plans (Table 3). According to the survey, the top three recommendations for improving preparedness differed slightly based on the type of organization. While a detailed sub-analysis was not possible due to the small sample size, respondents from faith-based and non-profit organizations suggested the need for more funding and resources, as well as better coordination between different organizations. On the other hand, healthcare centers and clinics suggested the need for better-executed and consistent policies, as well as standardized pandemic management protocols. Participants were given the option to report their recommendations in their own wording.

**Table 3:** Recommendations for better preparedness

Recommendations for better preparedness	Number of Organizations (*n* = 50)
Better coordination between all sectors (e.g., government organizations, health care, non-profit, etc.)	32 (64%)
Funding	28 (56%)
Consistent and better-executed policies and program plans	27 (54%)
Effective leadership communication	25 (50%)
Continuing education in disaster preparedness	24 (48%)
Standardized pandemic management protocol and pre-defined plans	20 (40%)
Other	1 (2%)

## Discussion

Our research findings contribute to the existing body of knowledge by highlighting the unequal distribution of social determinants of health in Baltimore through the use of geographic and survey data [16, 23–25]. Our results demonstrate that these disparities create varying levels of vulnerabilities and structural disadvantages among residents in different neighborhoods [16, 23–25]. Here, we focused primarily on the impact of the COVID-19 pandemic, the experiences, and the challenges faced in communities where vulnerabilities exist, in terms of social determinants of health, and where the burden of COVID-19 is the greatest. The use of COVID-19 Vulnerability index provided us with a simple measure to better understand the demographics, socioeconomic status, and health outcomes that are associated with vulnerable communities identified in this study. The analysis of geographic data highlighted the significant impact of social determinants of health on the health status, healthcare access, and COVID-19 vulnerability of Baltimore residents. These factors resulted in unique challenges reported by the participating organizations serving these communities, such as limited resources, lack of coordination, high levels of mistrust in the system, and misinformation.

Several studies described the incidence of communities with relatively high vulnerabilities that have significantly increased since the COVID-19 pandemic in several areas across the world [26–30]. Similarly, vulnerable communities identified in Baltimore City remained at greater risk during the pandemic. Our findings reveal substantial challenges faced by both vulnerable communities and the organizations that purposed to serve these communities during COVID-19. While nearly 80% of these organizations (surveyed) were actively providing services, funding was the leading challenge for most organizations. Without funding, this resulted in several limitations in properly serving vulnerable communities. For example, the populations served, reported experiencing several challenges during this pandemic including having access to essential resources. These findings were not surprising, since in many cases, the identified communities faced pre-existing challenges. For this reason, organizations played a significant role in providing necessities such as food and transportation; however, without this support of critical resources, communities were left at greater risk. Because of these limitations, a timely allocation and management of resources to vulnerable communities during the pandemic is critical to building some form of community resilience and lessening morbidity and mortality rates [31–33]. Previous studies show that having resources available eases the impact of major emergencies, aids in developing neighborhood resilience, and play a substantial role in better health outcomes [31, 34–38].

The lack of equal access to health care in vulnerable communities also contributed to increasing existing health inequalities [24, 37, 39]. With pre-existing health conditions, individuals in vulnerable communities were uniquely susceptible and disproportionately affected by the impacts of the COVID-19 pandemic. As of the date, Baltimore City communities were faced with the most cases, and the highest death rates in Maryland (Baltimore City Health Department, 2022). Populations served in this study experienced challenges in accessing COVID-19-related healthcare such as testing and treatment sites and healthcare insurance which are potential key factors leading to high death rates in Baltimore City. It is documented that at the beginning of the pandemic, the availability of COVID-19 testing sites did not always correspond with the vulnerability of the neighborhood, making them a hard access point for vulnerable populations, while they were more abundant near communities that were less vulnerable [40]. In many areas across the world, accessing health care, in general, during COVID-19 had its challenges due to health care systems being overburdened and under-sourced, though it was critical for communities with high-risk vulnerabilities [37, 39, 41].

During any crisis, the role of the government in communications, as well as the provision of accessible and accurate information, becomes critical more than ever [9, 34, 42]. For organizations that facilitated access to COVID-19-related information, having the access to accurate and up-to-date information themselves was quite challenging. Individuals’ need for information has increased significantly in time of big events such as the COVID-19 pandemic. Accessing updated and accurate information such as testing guidelines and eligibility, managing COVID-19, locating testing sites, transportation to testing sites, and where to find resources were key challenges for organizations providing services to vulnerable communities. In the early phase of the pandemic, government agencies and public health experts responsible for communicating information focused on engaging the public primarily to promote adherence to isolation and other protocols in an effort to stop the spread of the virus and fell short of providing additional pertinent information (Coronavirus Resource, 2020). In the later phases, public healthcare organizations begin partnering with community-based organizations (schools and churches) and healthcare centers to increase access to COVID-related information and resources. Having this pertinent information and access to health care is critical to developing community resilience in the face of disaster and in terms of rebounding afterward [36, 43]. Vulnerable communities, such as those identified in this study, would have more easily and positively navigated this emergency if there were better preparedness in terms of accessing and providing essential services.

Contributions to the increasing health inequalities and hardship seen in underserved vulnerable communities are primarily due to policy gaps and the lack of an equity perspective in the design and implementation of these practices [39, 44]. Increasing the awareness that the current policies in place are not effective and have failed to such support programs, can be strengthened, and tailored to aiding underserved communities [45]. This study highlights the unfavorable effects of policy programming related to living and working environments, particularly among vulnerable communities that previously suffer from food, housing, and job insecurities. Numerous challenges and insecurities in full effect prior to the pandemic coupled with the difficulties in accessing health services and other basic needs have exposed these individuals to the adverse effects of the pandemic [46–49]. Results from this study provide essential information for mandatory action for measures to improve preparedness and control of COVID-19 and other public health emergencies in vulnerable communities in the future. These findings can also be used to inform change to both health programming and policy. This information could potentially be utilized to improve and adapt new strategies that will help prepare local governments, community-based organizations, and community residents living in vulnerable areas for future public health disasters.

## Recommendations

Based on the challenges faced, organizations made the following recommendations for policy, practice, and programming for better preparedness. Nearly 65% of the organizations surveyed recommended having better coordination between all government organizations, health care, non-profit organizations, and others. The majority of these organizations served people who also reported having misinformation (disinformation around COVID-19) and faced challenges in terms of access to food, transportation, and COVID-related health care. Our research findings offer a valuable resource for developing targeted recommendations for public health strategies during public emergencies to improve health outcomes, particularly for vulnerable communities and specific types of organizations. These recommendations could include the development of better risk communication guides and plans, criteria for the allocation of funding and resources in high-risk areas, and increased involvement of community and organizational leaders in the decision-making process. Non-profit and faith-based organizations serving vulnerable communities require special attention and support, including specific criteria for resource allocation and funding. By implementing these strategies, we can work towards improving health equity and addressing the disparities exacerbated by social determinants of health. This approach will help to ensure that resources are effectively distributed and utilized to support those who need it most.

## Strengths and limitations

A strength of this study is that our questionnaire survey responses supported the knowledge and findings of historical challenges faced by vulnerable communities in Baltimore City. Another strength of this study is that we collected this information while people were still in the middle of the pandemic. Therefore, we obtained real-time insight into their experiences, which future studies will not be able to do. Our study also has a few limitations. One is that these findings are from a limited number of organizations in Baltimore City that provided services in response to COVID-19. Although we believe that our sample size of surveyed organizations is significant to inform city officials of the challenges faced by vulnerable communities and to provide insights for better preparedness, we also believe that there is a greater possibility that if all the organizations within these communities had surveyed, then responses may have been different. In addition, during COVID-19, we had to adhere to local COVID-19 guidelines and restrictions, and thus were not able to carry out in-person conversations and found it difficult to reach out to certain organizations who did not readily respond to our outreach methods. Given the nature of the chaotic response in the city, peoples’ time and attention were mostly absorbed by their role in the response, and we believe that this survey was not of priority to them. These limitations bring about restricted insights and approaches that have the potential to help better these community outcomes. With this in mind, we consider conducting a follow-up when things have calmed down, to try to capture the thoughts of some individuals who may have wanted to respond but were unable to because of these constraints.

## Conclusions

In a city that has been significantly affected by the COVID-19 pandemic, we have indicated needed ways to improve community health outcomes. This study suggests that in the case of disaster, vulnerable communities require access to essential resources and information to prepare in order to reduce their susceptibility to worse outcomes. The perspectives from several organizations involved in serving vulnerable populations demonstrated effectiveness in providing suggested recommendations that will help vulnerable communities with disaster planning (prepare and respond to) and will aid in building community resilience. We believe that developing partnerships across diverse disciplines and among health researchers, health care providers, community leaders, communities, community leaders and planners, and policymakers will be key to implementing these changes.

## Electronic supplementary material

Below is the link to the electronic supplementary material.

Supplementary Fig. 1. GIS Mapping of study sites in Baltimore City based on COVID-19 Community Vulnerability (CCVI) index. Sites are categorized by indicators (A) poverty rate, (B) unemployment rate percentile, (C) no high school diploma percentile, (D) non-White percentile, (E) lung cancer mortality rate, (F) heart disease mortality rate, G) sociodemographic vulnerability, and health vulnerability.

## Data Availability

The datasets analyzed during the current study were derived from the following public domain resources including Baltimore’s CSA data for the 2017 Neighborhood Health Profiles (https://health.baltimorecity.gov/neighborhoods/neighborhood-health-profile-reports); COVID-19 data (https://coronavirus.maryland.gov/); Zip code level data (https://opendata.maryland.gov/); Sociodemographic data (https://www.census.gov/programs-surveys/acs/data.html), and Spatial data (https://www.openstreetmap.org).

## Abbreviations

BTEC: Baltimore Transit Equity Coalition
GIS: geographic information systems
CBPR: community-based participatory research
CARES: Community-Aligned REsearch Solutions
CSAs: Community Statistical Areas
ACS: American Community Survey
CCVI: COVID-19 Community Vulnerability Index.
