# Supplementary material for: Lessons learned to improve COVID-19 response in communities with greatest socio-economic vulnerabilities
Source: BMC Public Health. 2023 Apr 6;23:659. doi: 10.1186/s12889-023-15479-0 (PMC10079160; doi:10.1186/s12889-023-15479-0)
Supplement: Supplementary file 1 — Supplementary Fig. 1. GIS Mapping of study sites in Baltimore City based on COVID-19 Community Vulnerability (CCVI) index. Sites are categorized by indicators (A) poverty rate, (B) unemployment rate percentile, (C) no high school diploma percentile, (D) non-White percentile, (E) lung cancer mortality rate, (F) heart disease mortality rate, G) sociodemographic vulnerability, and health vulnerability. [file 12889_2023_15479_MOESM1_ESM.docx]

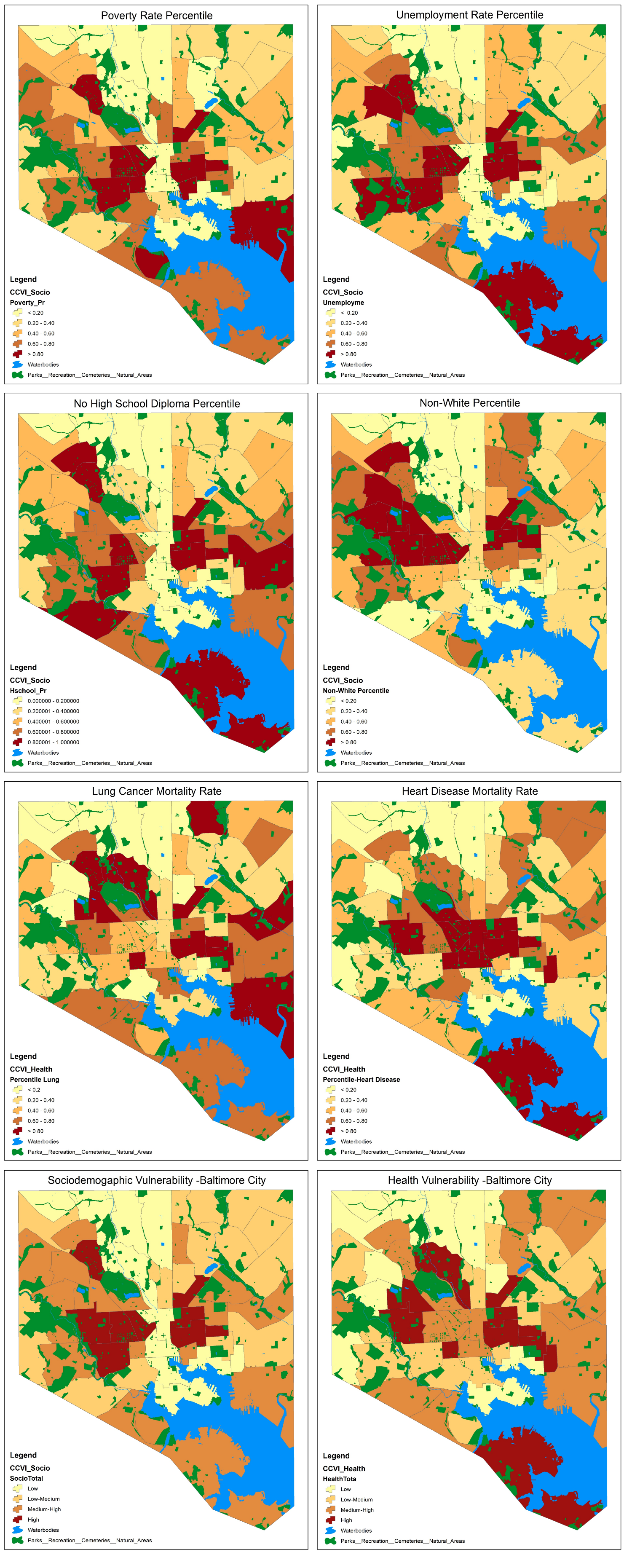


**A**

**B**

**C**

**D**


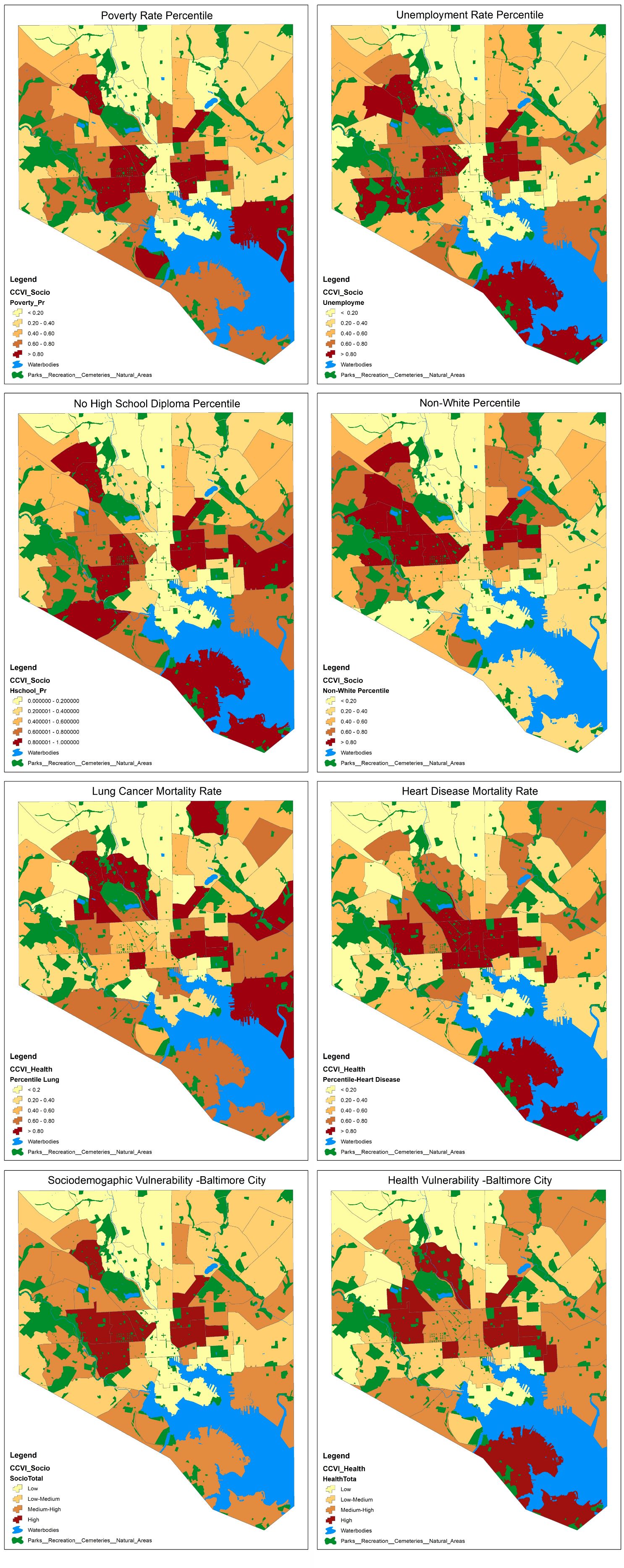


**E**

**F**

**G**

**H**

**Additional file 1. Supplementary Figure 1.** GIS Mapping of study sites in Baltimore City based on community covid vulnerability (CCVI) index. Sites are categorized by indicators (A) poverty rate, (B) unemployment rate percentile, (C) no high school diploma percentile, (D) non-White percentile, (E) lung cancer mortality rate, (F) heart disease mortality rate, G) sociodemographic vulnerability, and health vulnerability.
